# 4-Hydroxy-2-Nonenal-Modified Glyceraldehyde-3-Phosphate Dehydrogenase Is Degraded by Cathepsin G in Rat Neutrophils

**DOI:** 10.1155/2011/213686

**Published:** 2011-04-10

**Authors:** Yukihiro Tsuchiya, Go Okada, Shigeki Kobayashi, Toshiyuki Chikuma, Hiroshi Hojo

**Affiliations:** ^1^Department of Degenerative Neurological Diseases, National Institute of Neuroscience, National Center of Neurology and Psychiatry, 4-1-1 Ogawahigashi, Kodaira, Tokyo 187-8502, Japan; ^2^Department of Hygienic Chemistry, Showa Pharmaceutical University, 3-3165 Higashi-Tamagawagakuen, Machida, Tokyo 194-8543, Japan; ^3^Department of Analytical Chemistry of Medicines, Showa Pharmaceutical University, 3-3165 Higashi-Tamagawagakuen, Machida, Tokyo 194-8543, Japan

## Abstract

Degradation of oxidized or oxidatively modified proteins is an essential part of the antioxidant defenses of cells. 4-Hydroxy-2-nonenal, a major reactive aldehyde formed by lipid peroxidation, causes many types of cellular damage. It has been reported that 4-hydroxy-2-nonenal-modified proteins are degraded by the ubiquitin-proteasome pathway or, in some cases, by the lysosomal pathway. However, our previous studies using U937 cells showed that 4-hydroxy-2-nonenal-modified glyceraldehyde-3-phosphate dehydrogenase is degraded by cathepsin G. In the present study, we isolated the 4-hydroxy-2-nonenal-modified glyceraldehyde-3-phosphate dehydrogenase-degrading enzyme from rat neutrophils to an active protein fraction of 28 kDa. Using the specific antibody, the 28 kDa protein was identified as cathepsin G. Moreover, the degradation activity was inhibited by cathepsin G inhibitors. These results suggest that cathepsin G plays a crucial role in the degradation of 4-hydroxy-2-nonenal-modified glyceraldehyde-3-phosphate dehydrogenase.

## 1. Introduction

Low-to-moderate concentrations of reactive oxygen species affect a great number of physiological functions. However, when the reactive oxygen species concentration exceeds the antioxidative capacity of an organism, animal cells enter a state termed oxidative stress, in which the excess reactive oxygen species induces oxidative damage on cellular components. As a result, oxidative stress has been implicated in a large range of diseases, including cancer, diabetes, male infertility, autoimmune diseases, atherosclerosis, and cardiovascular disorders [1–3].

Exposure to oxidative stress, which occurs in the presence of reactive oxygen species and free radicals, causes many adverse events including modification of proteins and reactions with DNA [4]. Lipid peroxidation also occurs, and various reactive aldehydes, such as 2-alkenals, 4-hydroxy-2-alkenals, and ketoaldehydes, are generated [5]. 4-Hydroxy-2-nonenal (HNE) is a major reactive aldehyde formed by the peroxidation of *ω*-6 polyunsaturated fatty acids, such as linoleic acid and arachidonic acid [6], and is involved in various physiological and pathological reactions [5, 6]. 

HNE has a high stability and reactivity and is largely responsible for many types of cellular damage associated with oxidative stress. HNE covalently binds to cysteine, lysine, or histidine residues [5]. These adducts are detected in several disease lesions, including in the brains of patients with Alzheimer's disease [7, 8] and in low-density lipoproteins of atherosclerotic lesions [9]. HNE modifies enzymes and DNA and also affects protein synthesis and cell signaling [6, 10]. Many diverse enzymes are HNE sensitive, such as glyceraldehyde-3-phosphate dehydrogenase (GAPDH) (EC 1.2.1.12) [11], glucose-6-phosphate dehydrogenase [12], glutathione reductase [13], interleukin-1*β*-converting enzyme [14], and Na^+^, K^+^-ATPase [15].

The binding of HNE to proteins can cause conformational changes and amino acid modifications, as well as the accumulation and aggregation of proteins which causes cellular dysfunctions [16]. The breakdown of such modified proteins is an essential defense mechanism against oxidative stress in cells. The major proteolytic system for the degradation of oxidized or HNE-modified proteins is the ubiquitin-proteasome pathway [17–19]. Recently, ubiquitin-dependent lysosomal degradation of HNE-modified proteins has been reported in human lens epithelial cells [20].

GAPDH is a classical glycolytic enzyme which plays a central role in energy production. Mammalian GAPDH displays a number of diverse activities unrelated to its glycolytic function. These include its role in membrane fusion, microtubule bundling, phosphotransferase activity, nuclear RNA export, DNA replication, and DNA repair. Moreover, GAPDH is involved in apoptosis, age-related neurodegenerative diseases, prostate cancer, and viral pathogenesis [21]. Recent studies have established several new roles for GAPDH including transcriptional control of histone gene expression, recognition of fraudulently incorporated nucleotides in DNA, and its mandatory participation in the maintenance of telomere structure [22]. Furthermore, GAPDH plays an apparent role in Alzheimer's disease-related apoptotic cell death [23].

In previous reports, we indicated that GAPDH modified by HNE or by acetylleucine chloromethyl ketone, a synthetic inhibitor of acylpeptide hydrolase, is degraded enzymatically and is accompanied by the release of a 23 kDa fragment in the U937 leukemia cell line [24, 25]. Furthermore, we purified the HNE-modified GAPDH-degrading enzyme from a U937 cell extract and identified this enzyme as cathepsin G (EC 3.4.21.20) [26].

Neutrophils rapidly accumulate at sites of inflammation and infection, and these cells are activated by bacterial and host-derived factors. After phagocytosis of microorganisms or other particulate substances, neutrophils secrete a variety of mediators that possess potent proinflammatory and antimicrobial activities. These mediators include a group of antibiotic peptides and proteases that are stored in neutrophil granules and released during the process of degranulation. Human neutrophil elastase, proteinase 3, and cathepsin G are three serine proteases of the chymotrypsin family that are stored in the primary (azurophil) granules of neutrophils. Neutrophils form the first line of defense during infection and are indispensable in this function. However, imbalanced activity of neutrophil proteases may contribute to disease. This imbalance may result from insufficient control of neutrophil proteases, as well as from exposure to excessive protease activity due to the presence of increased numbers of neutrophils in inflamed tissues. Increased levels of neutrophil elastase are indeed associated with various inflammatory disorders such as chronic obstructive pulmonary disease, genital tract inflammation, and inflammatory bowel disease. Cystic fibrosis, a disorder characterized by a slow and relentless breakdown of the normal airway architecture, emphasizes the important contribution of neutrophil proteases to disease. Also, neutrophil proteases are associated with systemic vasculitis, such as Wegener's granulomatosis. This type of vasculitis can affect multiple organs including the lungs and kidneys and is characterized by granulomatous inflammation. Moreover, the majority of the patients with granulomatous inflammation also have circulating antineutrophil cytoplasmic autoantibodies directed against proteinase 3. Several reports indicate that such antibodies can activate neutrophils. Neutrophil proteases are also associated with tumor development and metastasis [27].

In the present study, we activated rat neutrophils with N-formyl-Met-Leu-Phe (fMLF). We then examined whether cathepsin G in the conditioned medium from these activated cells is effective in HNE-modified GAPDH degradation.

## 2. Results

### 2.1. Degradation of HNE-Modified GAPDH by Incubation with the Cell Extracts from Neutrophils

**Figure 1:**
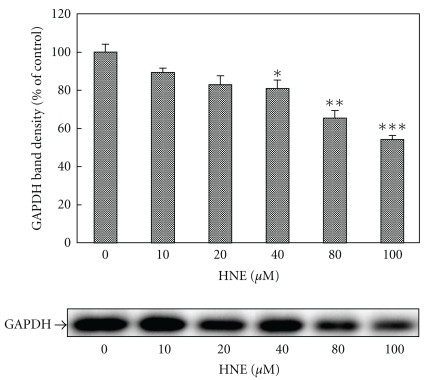
Concentration-dependent effect of HNE on degradation of GAPDH by incubation with cell extracts from neutrophils. The cell extract from neutrophils was incubated with eGAPDH at the indicated concentrations of HNE at 37°C for 3 h. Reaction products were separated on 12% SDS-PAGE gels and analyzed by Western blotting using an anti-GAPDH mAb. Data are mean ± SE (bars) values from four independent experiments. Significant decreases in GAPDH level compared to control are indicated by asterisks: *P < 0.05, **P < 0.01, and ***P < 0.001.

**Figure 2:**
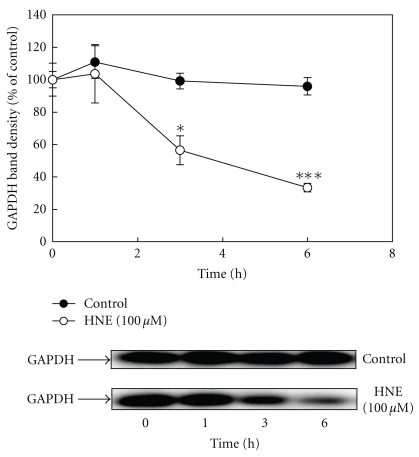
Time course of HNE-modified GAPDH degradation by incubation with cell extracts from neutrophils. The cell extracts from neutrophils were incubated with eGAPDH and 100 *μ*M HNE (empty circle) or with eGAPDH alone without HNE as a control (filled circle) at 37°C for the indicated times. Reaction products were separated on 12% SDS-PAGE gels and analyzed by Western blotting using an anti-GAPDH mAb. Data are mean ± SE (bars) values from four independent experiments. Significant decreases in GAPDH level compared to control are indicated by asterisks: *P < 0.05 and ***P < 0.001.

The dose-dependent degradation of GAPDH by HNE was examined using the cell extracts from neutrophils at concentrations from 10 to 100 *μ*M. As shown in Figure 1, the density of the band of GAPDH decreased with increasing doses of HNE. The time-dependent degradation of GAPDH by 100 *μ*M HNE was also examined. The density of the band corresponding to the 36 kDa band of GAPDH decreased over time. There was no decrease in the GAPDH level when the cell extracts from neutrophils were incubated without HNE (Figure 2).

### 2.2. The Separation of the GAPDH-Degrading Enzyme

**Figure 3:**
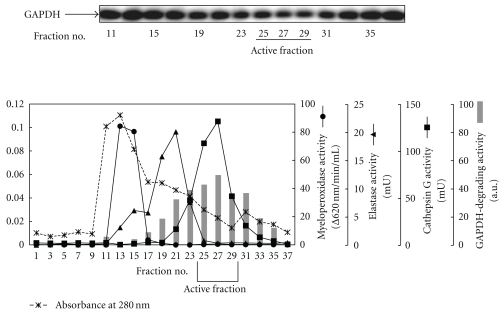
The gel filtration pattern of activity of the GAPDH-degrading enzyme in the cell extracts from neutrophils. The cell extracts from neutrophils were fractionated by Sephacryl S-200 HR column chromatography equilibrated with 50 mM sodium acetate buffer containing 1 M NaCl (pH 4.0). Activity of the GAPDH-degrading enzyme was assessed by the amount of the decreased GAPDH level, which was analyzed by Western blotting using an anti-GAPDH mAb and digitized immunoblots. Myeloperoxidase activity was determined spectrophotometrically. Elastase and cathepsin G activities were measured using fluorometric substrates.

Conditioned medium from rat neutrophils pretreated with cytochalasin B and fMLF was concentrated by ammonium sulfate precipitation and then fractionated on a Sephacryl S-200 HR column. The elution profile is shown in Figure 3. Since myeloperoxidase, elastase, and cathepsin G are released from neutrophils after stimulation with fMLF, activities of these enzymes were determined. Myeloperoxidase activity was detected at fractions 13–16 with the major protein peak. Cathepsin G and GAPDH-degrading activity were well separated from the major protein peak and coeluted in the same fractions. The active fraction 25–28 was used for further experiments.

**Figure 4:**
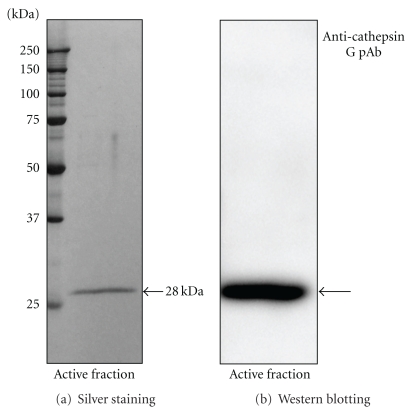
SDS-PAGE analysis and Western blotting of the active fraction separated from the cell extracts from neutrophils. The active fraction separated from the cell extract from neutrophils was analyzed by SDS-PAGE using 12% polyacrylamide gels. The protein bands were analyzed by silver staining (a) and Western blotting using an anticathepsin G pAb (b).

The result of SDS-PAGE of the active protein fraction isolated from the cell extracts from neutrophils is shown in Figure 4(a). The single band in the gel was at the molecular weight of 28 kDa. To identify the 28 kDa protein as cathepsin G, we used an anticathepsin G antibody in Western blots (Figure 4(b)).

### 2.3. Effects of Cathepsin G Inhibitors on the HNE-Modified GAPDH-Degrading Activity

**Figure 5:**
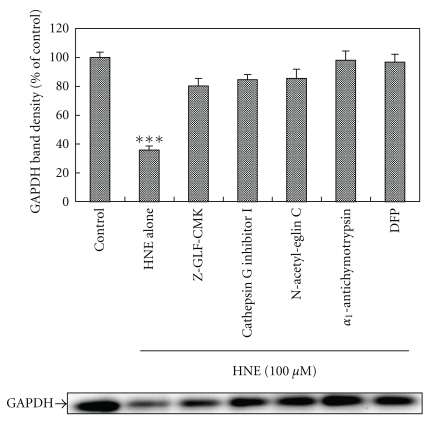
Effects of various cathepsin G inhibitors on the HNE-modified GAPDH-degrading activity of the active fraction. The active fraction separated from the cell extract from neutrophils was incubated with eGAPDH and 100 *μ*M HNE in the presence of DFP or various cathepsin G inhibitors (Z-GLF-CMK: 100 *μ*M; cathepsin G inhibitor I: 10 *μ*M; N-acetyl-eglin C: 1 *μ*M; *α*_1_-antichymotrypsin: 1 *μ*M; DFP: 1 mM) at 37°C for 3 h. The reaction products were separated by SDS-PAGE using 12% polyacrylamide gels and analyzed by Western blotting using an anti-GAPDH mAb. Data are mean ± SE (bars) values from four independent experiments. Significant decreases in GAPDH level compared to control are indicated by asterisks: ***P < 0.001.

The effects of various cathepsin G inhibitors on the HNE-modified GAPDH-degrading activity of the active fraction was investigated. The serine protease inhibitor DFP and cathepsin G inhibitors, Z-GLF-CMK, cathepsin G inhibitor I, N-acetyl-eglin C, and *α*_1_-antichymotrypsin, were used. The active fraction was incubated with eGAPDH and HNE in the presence of the respective inhibitors. All inhibitors efficiently inhibited the HNE-modified GAPDH-degrading activity of the active fraction (Figure 5).

## 3. Discussion

Neutrophils are the most abundant circulating leukocytes in humans and play a fundamental role in the innate immune response. This is best exemplified by patients with neutropenia, chronic granulomatous disease, or leukocyte adhesion deficiency syndrome, who are particularly prone to bacterial and fungal infections. Neutrophils are recruited rapidly to sites of inflammation. Their primary role is to kill invading bacteria and certain fungal species through phagocytosis by the release of preformed granular enzymes and proteins and by the production of a range of oxygen species. However, the highly destructive capacity of these cells also raises the potential for neutrophils to damage healthy tissues which occurs in many inflammatory diseases such as acute respiratory distress syndrome, inflammatory bowel disease, and rheumatoid arthritis [28, 29].

HNE is an *α*,*β*-unsaturated aldehyde that is formed by the peroxidation of *ω*-6 polyunsaturated fatty acids. HNE is increased in hypercholesterolemia and in atherosclerotic lesions causing accumulation of HNE-protein adducts [30]. Also, HNE promotes endothelial oxidative stress [10, 31], endothelial barrier dysfunction [32, 33], and apoptosis [34, 35]. Recently, several publications report the role of HNE-protein adducts in the pathogenesis and progression of Alzheimer's disease [36–38]. Since HNE-modified proteins cause many cellular dysfunctions, the removal of HNE-modified proteins causes important consequences for cell survival. However, the degradation mechanisms of HNE-modified proteins have not yet been clarified. Previously, we found that GAPDH degradation is triggered by HNE and 4-hydroxy-2-hexenal and is catalyzed by an enzyme that differs from proteasomes and lysosomal enzymes [25]. Moreover, we purified the HNE-modified GAPDH-degrading enzyme from a U937 cell extract by dialysis and sequential chromatography and identified this enzyme as cathepsin G [26].

Cathepsin G is a major serine protease found in the azurophil granules of neutrophils and monocytes and is abundant in U937 cells. Cathepsin G has various functions, including platelet activation, proteolysis of blood coagulation factors, angiotensin II generation, chemotactic activity on monocytes, and bactericidal activity. It is also involved in the degradation of myosin, elastin, proteoglycan, laminin, collagen, and immunoglobulins G and M [39, 40].

In the present study, we isolated an HNE-modified GAPDH-degrading enzyme from cell extracts of rat neutrophils by dialysis and Sephacryl S-200 HR column chromatography (Figure 3). As a result, an active fraction containing a 28 kDa protein was identified as cathepsin G using a cathepsin G-specific antibody (Figure 4). When the active fraction was incubated with eGAPDH and HNE in the presence of various cathepsin G inhibitors, the degradation of GAPDH was strongly inhibited (Figure 5).

We observed that HNE-modified GAPDH was also degraded in an extract of HL-60 cells, another human myeloblastic leukemia cell line, but not in an extract of Jurkat cells, a lymphoblastic leukemia cell line (data not shown). Together with the high expression of cathepsin G in neutrophils and monocytes, it is likely that the degradation of HNE-modified proteins by cathepsin G occurs selectively in granulocytes and monocyte cell types. It should be noted that the degradation of HNE-modified GAPDH by cathepsin G was independent of ubiquitin, whereas the degradation of HNE-modified proteins by proteasomes and lysosomal enzymes is dependent on ubiquitin [17, 18, 20]. 

**Figure 6:**
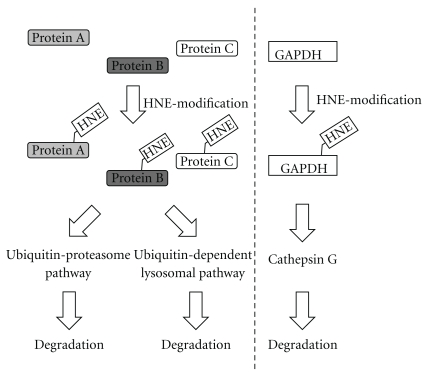
Mechanisms responsible for removing proteins modified by lipid peroxidation products. The major proteolytic system for the degradation of oxidized or HNE-modified proteins is the ubiquitin-proteasome pathway. Ubiquitin-dependent lysosomal degradation of HNE-modified proteins also has been reported in human lens epithelial cells. We observed that HNE-modified GAPDH was degraded by cathepsin G in granulocytes and monocytes.

Mechanisms responsible for removing proteins modified by lipid peroxidation products are not well understood. As a result, it is difficult to delineate the contribution of such a protein modification to disease severity or progression. Previous studies report conflicting results. In lens epithelial cells, HNE-modified proteins were preferentially ubiquitinated and degraded by the lysosome with little or no contribution by the proteasomal pathway of protein degradation [20]. In the kidney of ferric nitrilotriacetate-treated animals, proteasomal degradation was suggested as the crucial pathway for the removal of protein-HNE adducts [17]. A predominant role for the proteasome in the removal of protein-HNE adducts is, however, inconsistent with several studies showing that oxidative stress decreases proteasome activity and that HNE inhibits the proteasome by direct covalent modification [17, 41]. In addition, *in vitro* studies show that HNE-cross-linked proteins inhibit proteasomal activity [42], suggesting that protein degradation pathways other than the proteasome may be important for the removal of protein-HNE adducts. It is likely that different pathways of protein removal are engaged by different cells and that their contribution varies with the extent of lipid peroxidation [43] (Figure 6).

In conclusion, the present study has clarified that cathepsin G from rat neutrophils degrades HNE-modified GAPDH, suggesting that cathepsin G plays an important role in eliminating HNE-modified proteins formed during exposure to oxidative stress. 

## 4. Materials and Methods

### 4.1. Chemicals

Human erythrocyte GAPDH (eGAPDH), Z-Gly-Leu-Phe chloromethyl ketone (Z-GLF-CMK), N-acetyl-eglin C, and *α*_1_-antichymotrypsin were obtained from Sigma-Aldrich Co. (St. Louis, MO, USA). Diisopropyl fluorophosphate (DFP), cytochalasin B, and fMLF were purchased from Wako Pure Chemical Industries Ltd. (Osaka, Japan). Succinyl-Ala-Ala-Pro-Phe-4-methylcoumaryl-7-amide (Suc-AAPF-MCA) and N-methoxysuccinyl-Ala-Ala-Pro-Val-4-methylcoumaryl-7-amide (Suc(OMe)-AAPV-MCA) were obtained from the Peptide Institute (Osaka, Japan). HNE was from Cayman Chemical Co. (Ann Arbor, Mich, USA). Cathepsin G inhibitor I was from Calbiochem (Merck KGaA, Darmstadt, Germany). Sephacryl S-200 HR was from GE Healthcare Life Sciences (Piscataway, NJ, USA). Other chemicals and solvents were of analytical-reagent grade.

### 4.2. Antibodies

A mouse anti-GAPDH monoclonal antibody (mAb) was purchased from AbD Serotec (MorphoSys AG, Martinsried, Germany). A rabbit anticathepsin G polyclonal antibody (pAb) was purchased from Calbiochem. A biotinylated goat antimouse immunoglobulin pAb, biotinylated goat antirabbit immunoglobulin pAb, and horseradish peroxidase- (HRP-) conjugated streptavidin were obtained from Dako Denmark A/S (Glostrup, Denmark).

### 4.3. Isolation of Neutrophils and Preparation of Cell Extracts from Neutrophils

All animal experiments were carried out in accordance with the 1980 Animal Experiment Guidelines of the Japanese Government and have been approved by the Animal Experiment Committee of our university. Retired male Wistar rats were obtained from Sankyo Laboratory (Tokyo, Japan) and were housed with free access to food and water. A 12 : 12 light-dark cycle was maintained over 1 week. Neutrophils were isolated by polypeptone elicitation as described previously [44] with a minor modification. In brief, 5 mL of 10% polypeptone in sterile saline was injected intraperitoneally. Twelve hours later, the rats were anesthetized with diethyl ether and killed by decapitation. The peritoneum containing the neutrophils was rinsed with 30 mL of phosphate-buffered saline (PBS) containing 1 unit/mL of heparin. The rinsing solution was filtered through gauze and centrifuged for 7 min at 500 g. Red blood cells were lysed in 15 mL of 0.15 M NH_4_Cl.

To prepare the cell extract, neutrophils (2 × 10^8^ cells/mL) were collected, washed twice with ice-cold PBS, and treated with cell lysis buffer (20 mM phosphate buffer, pH 7.4, 1 mM EDTA, 0.05% (v/v) Triton X-100) for 3 min. The cell lysate was centrifuged at 17,000 g for 10 min at 4°C, and the resultant supernatant (cell extract from neutrophils) was used for further analysis. The protein concentration was determined by the Bradford method [45] using bovine serum albumin as a reference standard.

### 4.4. Purification Procedures for the HNE-Modified GAPDH-Degrading Enzyme

Neutrophils isolated from 14 rats were resuspended in Hanks' solution (3 × 10^9^ cells). Cytochalasin B and fMLF were added at final concentrations of 34.6 *μ*M and 6.6 *μ*M, respectively. The cells were incubated at 37°C for 1 h in a humidified atmosphere of 5% CO_2_ and 95% air and then centrifuged at 120 g for 15 min. The resultant conditioned medium was collected and subjected to ammonium sulfate (65% saturation) precipitation for 30 min. The precipitate was collected by centrifugation at 15,000 g for 30 min. The precipitate was resuspended in 4 mL of 50 mM sodium acetate buffer containing 1 M NaCl (pH 4.0) and dialyzed against the same buffer using a commonly used dialysis tube (molecular weight cutoff: 14,000). The minor precipitate was removed by centrifugation (8,000 g for 15 min). The supernatant fraction after dialysis was applied on a Sephacryl S-200 HR column (1.5 × 90 cm) equilibrated with the same buffer and eluted with the same buffer at a flow rate of 6 mL/h. The UV absorbance of the eluate was monitored at 280 nm, and column fractions (3.0 mL per tube) were assayed for elastase, myeloperoxidase, and cathepsin G activities.

### 4.5. Assay for the HNE-Modified GAPDH-Degrading Activity

An aliquot of each fraction was incubated with eGAPDH (6 *μ*g/mL) and 100 *μ*M HNE in 20 mM phosphate buffer (pH 7.4) containing 1 mM EDTA at 37°C for 3 h. To measure the degradation activity in the cell extract from neutrophils, an aliquot of the extract (0.4 mg/mL) was incubated with various concentrations of HNE at 37°C in the same buffer. The sample was then analyzed by Western blotting using the anti-GAPDH mAb.

### 4.6. SDS-PAGE and Western Blotting

Protein samples were prepared in SDS-PAGE sample buffer (62.5 mM Tris-HCl, pH 6.8, 4% (w/v) SDS, 20% (v/v) glycerol, 10% (v/v) 2-mercaptoethanol, and 0.01% (w/v) bromophenol blue). Aliquots of the samples were subjected to SDS-PAGE in 12% polyacrylamide gels according to the method of Laemmli [46]. For silver staining, 12% polyacrylamide gels were stained using a Wako Silver Stain II Kit (Wako Pure Chemical Industries Ltd.). For Western blotting, gels were electroblotted onto a polyvinylidene difluoride membrane (Immobilon-P Transfer Membrane; Millipore Co.) at 180 mA for 2 h using a semidry transfer system (Trans-Blot SD Cell; Bio-Rad Laboratories Inc., Hercules, Calif, USA). The blotted membrane was blocked in 2% Block Ace Solution (Dainippon Sumitomo Pharma Co. Ltd., Osaka, Japan) for 1 h, probed with the mouse anti-GAPDH mAb (16 ng/mL) or rabbit anticathepsin G pAb (0.5 *μ*g/mL) for 2 h, and then visualized using the biotinylated antimouse or antirabbit immunoglobulins pAb, HRP-streptavidin, and Immobilon Western Chemiluminescent HRP Substrate (Millipore Co.). A LAS-1000 UV mini F85 Luminoimage Analyzer (Fujifilm Co., Tokyo, Japan) was used for chemiluminescence detection. The density of each band was quantified by Multi Gauge software (Version 3.0; Fujifilm Co.) and expressed in arbitrary units.

### 4.7. Assays for Enzyme Activities

Cathepsin G and elastase activities were measured by known methods using Suc-AAPF-MCA and Suc(OMe)-AAPV-MCA as substrates, respectively [47, 48]. The fluorescence intensity of the solution was read at an emission wavelength of 460 nm and an excitation wavelength of 360 nm. Activity of myeloperoxidase was determined spectrophotometrically (620 nm) using tetramethylbenzidine and H_2_O_2_ as substrates [49]. One unit of enzyme activity is defined as the amount of enzyme required for 1 *μ*mol of the substrate to turn into the corresponding product in 1 min at 37°C.

### 4.8. Statistical Analysis

Data are represented as the mean ± SE and analyzed by a two-tailed unpaired Student's *t*-test.

## Abbreviations

DFP: Diisopropyl fluorophosphate
eGAPDH: Erythrocyte glyceraldehyde-3-phosphate dehydrogenase
fMLF: N-formyl-Met-Leu-Phe
GAPDH: Glyceraldehyde-3-phosphate dehydrogenase
HNE: 4-hydroxy-2-nonenal
HRP: Horseradish peroxydase
mAb: Monoclonal antibody
Suc(OMe)-AAPV-MCA: N-methoxysuccinyl-Ala-Ala-Pro-Val-4-methylcoumaryl-7-amide
pAb: Polyclonal antibody
PBS: Phosphate-buffered saline
Suc-AAPF-MCA: Succinyl-Ala-Ala-Pro-Phe-4-methylcoumaryl-7-amide
Z-GLF-CMK: Z-Gly-Leu-Phe-chloromethyl ketone.
